# Probabilistic MRI Tractography of the Optic Radiation Using Constrained Spherical Deconvolution: A Feasibility Study

**DOI:** 10.1371/journal.pone.0118948

**Published:** 2015-03-05

**Authors:** Jeremy C. Lim, Pramit M. Phal, Patricia M. Desmond, Andrew D. Nichols, Chris Kokkinos, Helen V. Danesh-Meyer, Andrew H. Kaye, Bradford A. Moffat

**Affiliations:** 1 Department of Radiology, The University of Melbourne, Victoria 3050, Australia; 2 Department of Radiology, The Royal Melbourne Hospital, Grattan Street, Parkville, Victoria 3050, Australia; 3 Department of Surgery, The University of Melbourne, Victoria 3050, Australia; 4 Department of Neurosurgery, The Royal Melbourne Hospital, Grattan Street, Parkville, Victoria 3050, Australia; 5 Department of Ophthalmology, The University of Auckland, Auckland, New Zealand; University of Maryland, College Park, UNITED STATES

## Abstract

**Background and Purpose:**

Imaging the optic radiation (OR) is of considerable interest in studying diseases affecting the visual pathway and for pre-surgical planning of temporal lobe resections. The purpose of this study was to investigate the clinical feasibility of using probabilistic diffusion tractography based on constrained spherical deconvolution (CSD) to image the optic radiation. It was hypothesized that CSD would provide improved tracking of the OR compared with the widely used ball-and-stick model.

**Methods:**

Diffusion weighted MRI (30 directions) was performed on twenty patients with no known visual deficits. Tractography was performed using probabilistic algorithms based on fiber orientation distribution models of local white matter trajectories. The performance of these algorithms was evaluated by comparing computational times and receiver operating characteristic results, and by correlation of anatomical landmark distances to dissection estimates.

**Results:**

The results showed that it was consistently feasible to reconstruct individual optic radiations from clinically practical (4.5 minute acquisition) diffusion weighted imaging data sets using CSD. Tractography based on the CSD model resulted in significantly shorter computational times, improved receiver operating characteristic results, and shorter Meyer’s loop to temporal pole distances (in closer agreement with dissection studies) when compared to the ball-and-stick based algorithm.

**Conclusions:**

Accurate tractography of the optic radiation can be accomplished using diffusion MRI data collected within a clinically practical timeframe. CSD based tractography was faster, more accurate and had better correlation with known anatomical landmarks than ball-and-stick tractography.

## Introduction

The optic radiation (OR) is a fan-like white matter structure which originates in the lateral geniculate nucleus (LGN) and terminates posteriorly in the calcarine sulcus of the occipital lobe. It is essential for transmission of visual information between the primary visual cortex (V1) [1,2] and the LGN. *In vivo* visualization of the OR has been made possible with the development of diffusion weighted imaging (DWI) [3] and its associated tractography techniques. Accurate non-invasive visualization and segmentation of the OR in individual patients has tremendous potential for clinical translation, particularly in the area of pre-surgical planning for patients with temporal lobe epilepsy [4,5] and brain tumors. In addition, information about OR structure and integrity derived from tractography data has potential as a non-invasive biomarker for studying axonal integrity in diseases that affect the optic pathway, such as optic neuritis, multiple sclerosis and compressive tumors.

Previous attempts to visualize the OR using diffusion-based tractography have had mixed results [4–16]. The most anterior section of the OR, Meyer’s loop, remains a challenge to define due to the sharp angulation and reduced fiber density in this area compared to the body of the OR. These factors predispose tractography algorithms to terminate in this region, or to generate erroneous tracts. As such, the estimated anterior position of Meyer’s loop provides a useful indicator of the relative performance of different OR tractography methods [9,10,13–15,17]. To date, there is a paucity of comparative studies of DWI tractography techniques, particularly in the visualization of difficult white matter structures necessary for the clinical translation of diffusion tractography. In addition, many prior studies [6,7,18] have utilized acquisition protocols with a large number of diffusion sensitizing directions which allow for sophisticated diffusion modeling and subsequently better tractography results. The disadvantage of this approach is the long acquisition time (between 10–30 minutes). For tractography to be applicable as a clinical tool the acquisition time must be within tolerable limits for the patient so that it can be incorporated into existing clinical MRI protocols.

With this in mind, the purpose of this study was to investigate imaging the OR using probabilistic tractography [19] based on DWI data from a clinically feasible acquisition protocol [20] where either constrained spherical deconvolution (CSD) [21] or the ball-and-stick (B&S) model [19] was used to estimate the local fiber orientation distributions (FOD) in each voxel. The CSD calculations inherently allow crossing, kissing and bending fibres to be accounted for, whereas B&S assumes a model of varying complexity, requiring a non-linear fit of diffusion data for calculating the FOD.

The hypothesis was that 30 direction DWI data collected in less than 5 minutes would be sufficient to allow probabilistic tractography algorithms [19,21] to accurately depict the OR, in particular Meyer’s loop. If proven to be successful, such a protocol could be incorporated into the pre-surgical planning and follow up MRI scans of patients with pathology directly affecting or in the vicinity of the optic pathway.

There are theoretical advantages to both CSD [21] and B&S [19] estimates of the FODs necessary for probabilistic tractography, but to our knowledge they have yet to be compared for tracking the OR. Therefore, a second aim was to assess the performance of CSD compared to B&S based tractography in terms of computational times, quantitative receiver operating characteristic (ROC) analysis [6], and correlation to previous OR tractography studies [9,10,13–15,17]. Our hypothesis was that because CSD provides a framework to efficiently model complex fiber distributions, it would improve the computational times, sensitivity and accuracy of probabilistic OR tractography.

## Methods

### Ethics Statement

This study was approved by the Melbourne Health Human Research Ethics Committee. All imaging data was obtained retrospectively from The Royal Melbourne Hospital imaging archive, anonymized and de-identified prior to analysis.

### Subjects

Twenty patients who underwent clinically-indicated (see S1 Table) MRI of the brain at The Royal Melbourne Hospital had additional DWI data acquired suitable for probabilistic tractography based on both CSD and B&S FODs. Patients with significant midline shift, lesions in the parieto-occipital region or significant vision impairment were excluded from the study.

### Data Acquisition

Standard single-shot spin-echo echo planar imaging [22] on a Siemens Trio 3T (Erlangen, Germany) MRI scanner was used to acquire the DWI data sets. Whole brain coverage was achieved with 55 contiguous 2.5 mm thick axial slices a time (TR) of 8600ms and echo time (TE) of 120ms. Diffusion sensitizing gradients were applied in 30 non-collinear directions [23] (b-value = 3000s/mm^2^) using a diffusion weighted echo planar sequence with a parallel imaging factor of 2. The field of view was 240mm, acquisition matrix size 96 x 96, and the voxel size was 2.5x2.5x2.5 mm. The acquisition time for the DWI scans was 4 minutes and 26 seconds per subject. All 20 patients had additional anatomical scans performed, which included at least one 3D volumetric MRI acquisition (19 T1 weighted and 1 FLAIR weighted).

### Pre-Processing, ROI Generation and Tractography

All imaging data (DICOM format) was converted into accessible file formats for use in the FSL (Oxford, UK, http://www.fmrib.ox.ac.uk/fsl/) and MRtrix (Melbourne, Australia, http://www.brain.org.au/software/) tractography software packages. In addition, the MNI152 standard brain and Jülich probabilistic atlas [24,25] contained within FSL were used to process and analyze the OR tractography. The Jülich atlas is a map of brain structures where the intensity of each voxel is a measure of the probability that the voxel represents a particular structure based on the post-mortem dissection of ten brains [26]. The anatomical scans and MNI152 standard brain (including OR, LGN and V1 probability maps) were registered and resampled to the DWI co-ordinate space using a 12 parameter affine registration (FSL) [27] to the non-diffusion weighted image.

Based on diffusion tensor imaging (DTI) theory [3] the mean apparent diffusion coefficient (ADC), fractional anisotropy (FA), eigenvalue and eigenvector maps were calculated in FSL (using dtifit command) to allow basic inspection of the DWI data quality and to confirm that FSL had correctly imported the data. Also within FSL, bedpostX [19] was used to calculate FODs based on the B&S model. Using MRTrix, maps of the FODs were calculated using CSD [21] with a maximum harmonic order of 6 (CSD algorithm).

**Fig 1:**
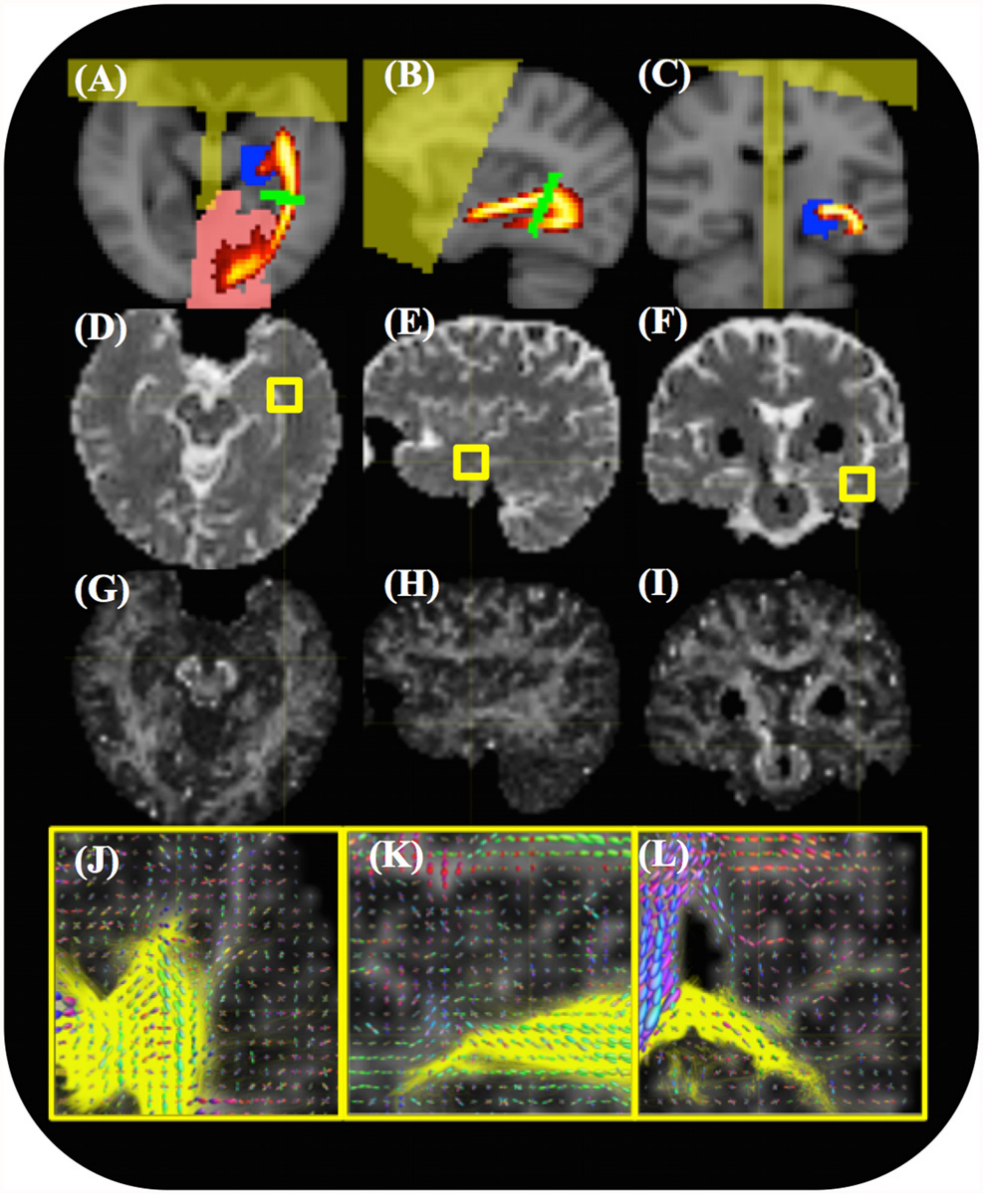
An example of the input data and ROIs used for fiber tracking. The seeding/target ROIs are overlaid on registered anatomical images. (A-C) ROIs in the axial, sagittal and coronal planes include the LGN seed (blue), V1 target (red) and waypoint (green) areas, a frontal/midline exclusion zone (yellow) and an atlas-defined OR probability map (red/yellow) overlaid on the MNI152 standard brain. (D-F) Representative axial, sagittal and coronal slices of the ADC map. (G-I) Representative axial, sagittal, and coronal slices of an FA map. (J-L) FOD plots for each voxel and the optic radiation (yellow) overlaid on the FA map in the region of Meyer’s loop indicated by the cross-hairs in (G-I).

To seed, target and analyze the tractography, binary masks of the OR, LGN and V1 were created by thresholding the probability maps such that only voxels belonging to more than one patient were retained [6]. The OR mask was also used to create a waypoint mask on a coronal slice 60 mm posterior to the temporal pole (TP) [6], an exclusion coronal plane 20mm posterior to the TP and a midline sagittal termination plane (Fig. 1). A white matter inclusion mask was created by thresholding each hemisphere of the FA map at 0.1.

All tractography was computed on a 12 core Intel based workstation (Hewlett Packard, CA, USA). MRtrix was configured to use 2 cores, while FSL was configured to use only a single core. The LGN mask (above) was used to seed tractography targeted to the V1 mask with a single coronal slice waypoint at the midpoint of the OR (Fig. 1). This is the similar to the approach used by Yamamoto et al. [13], except in our study all three ROIs were generated automatically by registration to the Jülich atlas. Tractography was performed using probabilistic algorithms contained within both the FSL (probtrackX) [19] and MRTrix [28] packages to calculate 5000 streamlines per seed voxel with a 0.2mm step length, a 0.3mm radius of curvature cutoff and an FA threshold of 0.1. Only those streamlines that passed through the waypoint mask to the V1 mask, and not entering the exclusion zone were retained. Both MRTrix and FSL allowed for conversion of these streamlines into 3D streamline density images (SDI). These had the image dimensions of the DWI acquisition and voxel intensities that represented the number of streamlines passing through it normalized to the total number of streamlines retained.

### Post-Processing and Analysis

The resulting SDIs were then post-processed and analyzed using MATLAB (Boston, USA). An ROC analysis [6] was implemented to quantitatively compare the tractography algorithms using the OR probability map of the Jülich atlas as a “gold standard”. Individual ROC plots were constructed by calculating the sensitivity or true positive rate (TPR) at 1000 equally spaced false positive rates (FPR) between 0 and 1. The TPR was defined as the proportion of retained SDI voxels which was contained within the OR binary mask. The FPR was defined as the proportion of retained SDI voxels which were outside of the OR binary mask but within the tractography inclusion mask. The area under the ROC curve (AUC) and Youden’s index (*J*) [29] were calculated for each individual SDI.

To calculate the final thresholded SDIs, an FPR threshold of 2.1% across all subjects was chosen as it was the median FPR previously found to best match probabilistic tractography of the optic radiation to the reference atlas [6]. For each patient the SDI intensity was calculated at this threshold. The median of this threshold was then used to create the B&S and CSD binary OR tracts for each patient. Anatomical locations, including the TP, occipital pole (OP) and the anterior tip of Meyer’s loop (MLA), were identified by an attending Neuroradiologist (PP) on the final thresholded SDIs overlaid on the anatomical images. The distances from MLA to TP and OP to TP resulting from both tractography methods were compared statistically against results from a dissection study [2] and against previous diffusion tractography studies [9,10,13–15,17].

### Statistical Analysis

Statistical analysis was conducted using the Statistical Toolbox contained within MATLAB (Boston, USA). The sensitivity, FPR, *J* and AUC of the CSD tracking algorithm were compared against their B&S counterparts using a paired sample Wilcoxon signed rank test. This statistical test was chosen because each of these measures cannot be assumed to be normally distributed. The MLA-TP distances were also compared using a paired sample t-test.

## Results

The 4 minute 26 second scan was well tolerated by all subjects. No significant motion artifact was present in any of the DWI images. FSL reconstructions and tractography took 3–4 hours to execute per subject, while processing of the CSD reconstructions took 1–2 hours per subject. The location of the ROIs used to seed, target and confine the tractography are shown in Fig. 1A–C super imposed on T1 weighted images. The quality of the ADC (Fig. 1D–F) and FA (Fig. 1G–I) maps was generally very good with no obvious artifacts in any of the images. On focused assessment of Meyer’s Loop on the FOD maps (Fig. 1J–L) it was possible to visualize the complexity of crossing and bending fiber bundles in this area.

### Tractography

**Fig 2:**
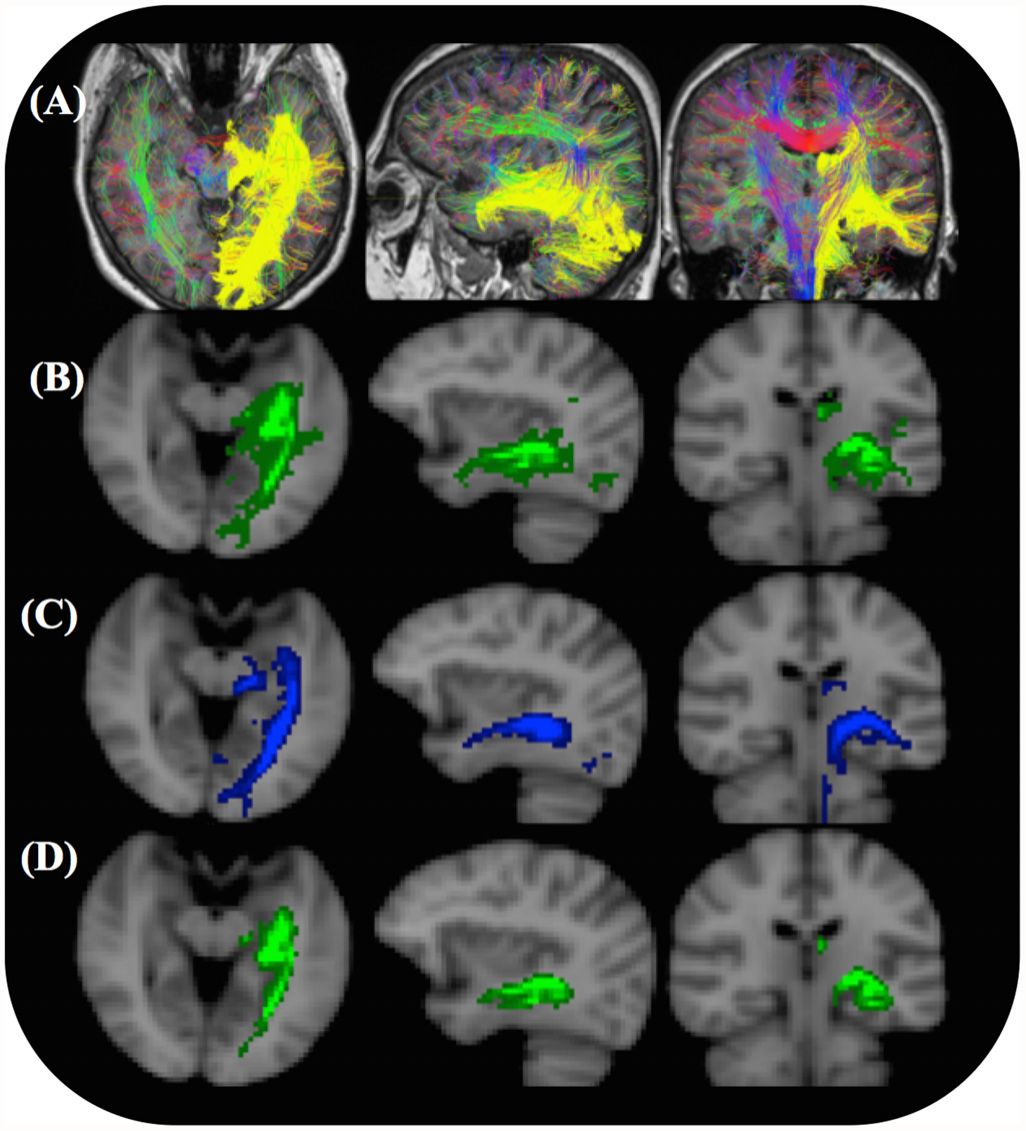
Complete CSD and B&S (probtrackX) tractography results for a single subject. (A) Without using a threshold to remove voxels of low connectivity. The unthresholded CSD streamline reconstructions of the OR (yellow) in three planes are shown together with whole brain color-coded CSD tracks for visual reference. (B) The unthresholded B&S SDIs (green) overlaid on anatomical T1 weighted images in three planes. (C) Final thresholded CSD SDI. (D) Final thresholded B&S SDI. An optimal threshold (Table 1) was used to remove voxels of low connectivity such that the median FPR across all patients was 2.1%.

The OR was reconstructed in all 20 subjects using both tractography algorithms. An example of the raw tractography results for a single patient is shown in Fig. 2A–B. The general shape of these resulting OR tracts and SDIs were consistent with the shape of the OR in the probability atlas (Fig. 1A–C). In addition, the tractography using the CSD algorithm (Fig. 2) was significantly more sensitive than with the B&S algorithm (Table 1). However, in all cases there was a significant number of voxels containing reconstructed OR streamlines that were most likely not part of the individual’s OR (false positive voxels).

**Table 1:** Tractography results and ROC analysis compared to previous studies.

	CSD	B&S	Dissection [2,4]	Previous tractography [4]
Tracks initiated	1494500	1494500		
Tracks retained	22350 (1800)	10893 (5000)		
Youden Index, *J*	0.61* (0.05)	0.41 (0.07)		
AUC	0.87* (0.03)	0.71 (0.04)		
Sensitivity, no threshold (%)	91.0* (7)	47 (8)		
FPR, no threshold (%)	36* (10)	7 (3)		
Sensitivity at *J* (%)	80 (5)*	47 (8)		
FPR at *J* (%)	18 (4)*	7 (3)		
Final tract threshold (%)#	0.45	0.02		
Final tract Sensitivity (%)	35 (6)*	23 (5)	42 (6)	30 (20)
Final Tract FPR (%)	2.1 (0.7)	2.1 (0.7)	N.A.	2.1 (1.6)
Final Tract volume (cm3)	28 (6)	21 (4)	18 (2)	16 (8)

Values shown are medians over 20 subjects, with standard deviations shown in brackets.

# This is the minimum SDI intensity normalized to the total number of streamlines retained by the tractography algorithm.

* p < 0.001 for a statistically significant difference compared to B&S method, based on a Wilcoxon signed rank test (see Statistical Analysis).

### Post Processing and Analysis

**Fig 3:**
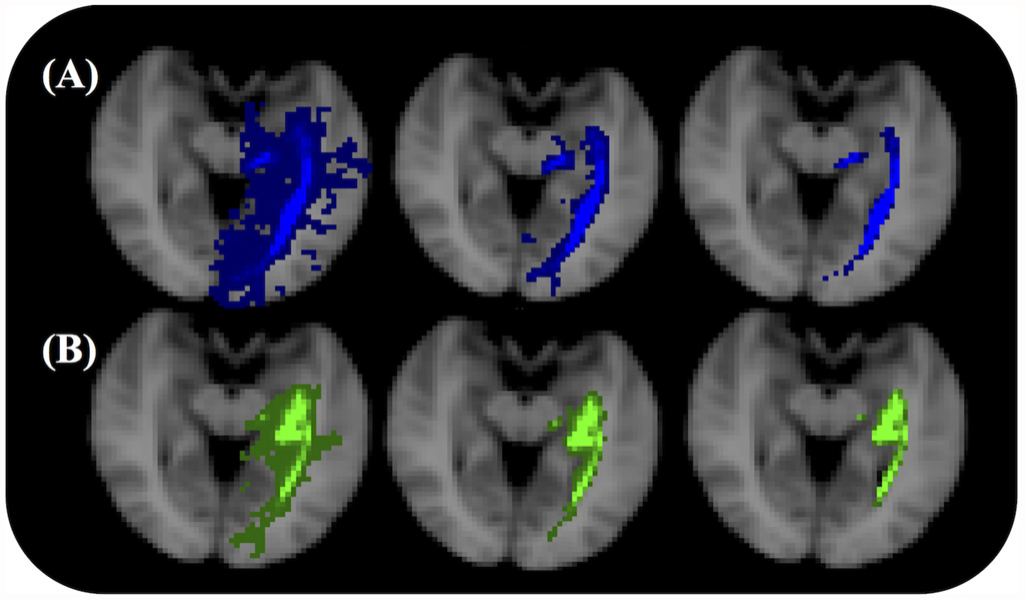
Left optic radiation SDIs for a single subject. The OR of the same subject as in Fig. 2 is shown with no threshold (left), the optimal SDI threshold (middle) and 10 times the optimal SDI threshold (right). (A) SDIs based on the CSD model. (B) SDIs based on the B&S model.

**Fig 4:**
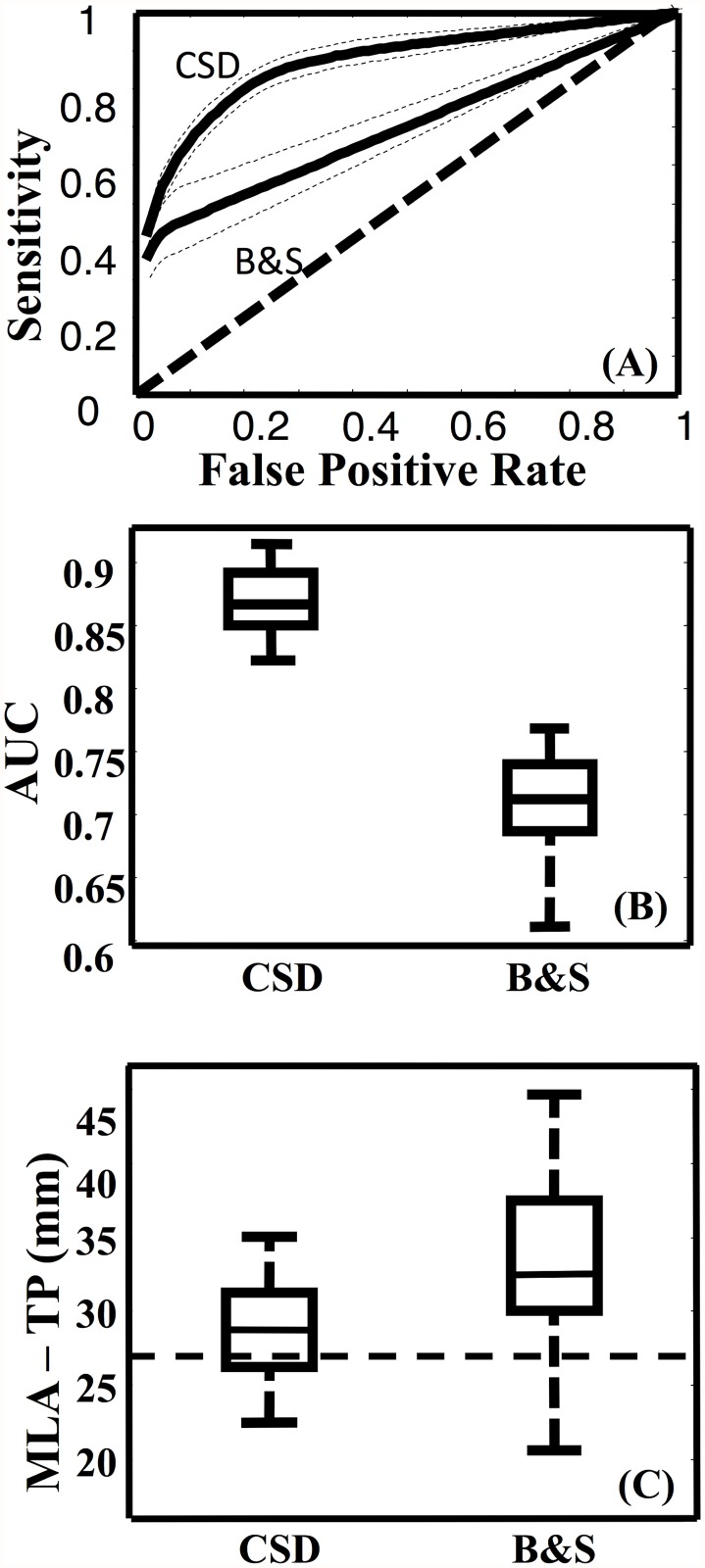
Group results of optic radiation fiber tracking. (A) The mean ROC curves with 95% confidence intervals (dashed lines) for the CSD and B&S based tractography. A line of unity is shown for comparison. (B) Box plot of the AUCs for the CSD and B&S tractography algorithms. The CSD algorithm resulted in a significantly (p<0.05) greater AUC than the B&S algorithm. (C) Box plots of the MLA-TP distances as calculated from the CSD and B&S thresholded streamline density maps. The dashed line indicates the median distance calculated from a previous dissection study [2].

To conduct the ROC analysis, the binary OR mask, which included Meyer’s loop (MLA to TP distance of 27 mm) was used. The mean search volume was 640 cm^3^ which included the union of all OR tracts and the atlas OR mask. Fig. 3 shows the SDIs from the two tractography algorithms with three different minimum thresholds. When this threshold was increased the OR became thinner and Meyer’s loop was observed to be more posterior. The mean ROC curves (and confidence intervals) for each algorithm are shown in Fig. 4A. The AUCs (Fig. 4B), Youden indices, sensitivities and FPRs were all significantly higher for the results of the CSD algorithm than those resulting from the B&S model (p < 0.001) (Table 1).

To calculate the final OR SDIs (Fig. 2C and D) false positive voxels were removed from the SDIs using a threshold that resulted in the same FPR as a previous study [6]. The thresholds used were small compared to the number of streamlines retained in each count image (less than 0.5%). For both algorithms the median volume (Table 1) of the OR was within a standard deviation of the inter quartile range found from the dissection study [24] used to create the probabilistic atlas.

After thresholding to calculate the final SDIs (Fig. 2C and D), the CSD method was still significantly more sensitive than the B&S method (p < 0.01) for reconstructing the OR. At the optimized threshold the median FPRs of the two algorithms were consistent with those of a previous study [6]. However, only the sensitivity of the CSD algorithm was consistent with the sensitivity of the individual ORs used to construct the histological reference atlas (Table 1).

### Anatomical Measurements

The distances measured between MLA and the anatomical landmarks were in good agreement with dissection results by Ebeling and Reulen [2] and previous OR tractography studies [9,10,13–15,17]. All measurements are summarized in Table 2. The distance between the two anatomical landmarks (TP and OP) were measured and compared to previously reported [2] dissection results. The mean TP to OP distance was 119 ± 7.6 mm (range = 108–133 mm), which was in accordance with dissection results (125 ± 4.7 mm, range = 116–134 mm). The mean MLA-TP distances (Fig. 4c) were significantly (p<0.01) shorter when based on the CSD tracts (30 ± 4 mm) compared to the B&S tracts (35 ± 7 mm).

**Table 2:** Distances between the anterior tip of Meyer’s loop and anatomical landmarks.

Measurement	MLA-TP distance *X* ± *SD* (min-max)	MLA-OP distance *X* ± *SD* (min-max)
CSD*	30 ± 4 (20–34)	92 ± 6.9 (81–103)
B&S	35 ± 7 (23–45)	87 ± 5.5 (76–96)
Dissection [2]	27 ± 3.5 (22–37)	98 ± 6.2 (85–108)
Sherbondy et al. [10]	28 ± 3.0 (24–34)	96 ± 5.5 (89–108)
Yamamoto et al. [13]	37 ± 2.5 (33–40)	82 ± 3.0 (77–85)
Wu et al. 2012 [15]	40 ± 3.8 (35–50) Left 41 ± 5.7 (35–54) Right	87 ± 5.5 (79–95) Left 86 ± 5.4 (80–94) Right
Taoka et al. [9]	37 ± 4.6 (30–43)	N/A
Nilsson et al. [17]	44 ± 4.9 (34–51)	N/A
White and Zhang 2009 [14]	31 ± 0.6 (26–33) Left 31 ± 1.7 (29–34) Right	N/A

MLA = Anterior tip of Meyer’s loop

TP = temporal pole

OP = occipital pole. All measurements are means ± SD (mm).

* = p < 0.01 for a statistically significant difference in MLA—TP distance compared to B&S method, based on a paired sample t-test (see Statistical Analysis).

## Discussion

The acquisition of DWI data using 30 non-colinear diffusion sensitizing (b = 1000) gradients allows data of sufficient quality to be obtained in a time frame that is clinically feasible [20]. Therefore, we have used this acquisition time (less than 5 minutes) to conservatively constrain our DWI acquisition scheme. Increasing magnitude of the diffusion weighting (b = 3000) does, however, significantly [19,21] improve the angular resolution in both the B&S and CSD models of crossing fibers. This is mainly due to an increase in the contrast-to-noise ratio [19,21,28] in the angular domain of the crossing fibres, despite the individual diffusion weighted images having a lower signal-to-noise ratio. While the number of diffusion weighted images may not be ideal, this study has shown that probabilistic tractography of the OR with this quantity of diffusion data is feasible. The long processing times (approximately 1–2 hours using the CSD algorithm and 4 hours using B&S algorithm) do limit the current clinical potential to non-emergency neurosurgical planning. However, it is expected that these processing times will decrease significantly as software packages take advantage of the ever improving computational power offered by advances in computer technology. While software efficiency cannot be ruled out, we believe that the main differences in computation time are due to the different approaches taken to estimating the FODs [19,21] and slight differences [19,28] in the way FODs are randomly sampled during the track calculations.

With respect to tractography of the OR, some important considerations are the choice of seed, target, waypoint, inclusion, termination and exclusion masks for initiating the tractography algorithm [16]. The advantages of using an automated method are that these areas are rapidly computed and there is no inter-observer variability in the generated ROIs. Nevertheless, these methods have shortcomings which stem from the fact that they extract ROIs from a probabilistic atlas and rely on registration of these ROIs to DWI space. The registration methods are susceptible to errors caused by inter-subject variation [30]. In addition, their utility may be compromised if these regions are displaced by pathology such as brain tumors. To address these issues, the ROIs were created to be substantially over-inclusive, resulting in a lack of initial specificity for the OR. Previous studies have shown that probabilistic algorithms have difficulty tracking Meyer’s loop if the seed voxels are placed directly at the LGN [7,16]. Many studies attempt to circumvent this problem by manually identifying the OR (based on the FA map) as it exits the LGN to be used as the seed ROI [7,16–18,31,32]. Other studies have seeded from the optic chiasm and/or Meyer’s Loop [14,16]. Manually placing seed points in this way is time consuming, highly subjective, and a potential source of error [30,32].

Both algorithms used in this study were able to identify Meyer’s loop in at least 19 out of 20 subjects. The estimated mean anterior position of Meyer’s loop differed by less than 1 cm from that reported in the largest dissection study based on Klingler’s fiber dissection technique (27 mm posterior to the temporal pole) [2,33]. Other dissection studies [12,34] report the mean MLA—TP distance as 25 mm and 31 mm respectively. In a study [24] where myelin-stained histological sections were matched to MR sections of the same brain the mean MLA—TP distance was found to be 23 mm, supporting the results of the dissection studies. Using the measurements above as a gold standard, it can be concluded that the tracking methods used in this study perform as well as, or better than, those in previous tractography studies. For example, a study [7] on 41 subjects using probabilistic tractography (52-direction DTI) and manual ROI segmentation reported the mean MLA—TP distance to be 34 mm. Another study correlating visual field defects (VFDs) after temporal lobectomy with the anterior limits of Meyer’s loop (estimated by DTI) reported a mean MLA—TP distance of 32 mm [8]. Studies which rely on deterministic algorithms estimate Meyer’s loop to be located even more posteriorly [9,13,17] (Table 2). Thus, although the computational simplicity of deterministic algorithms and ease of interpretation is highly attractive for clinical applications, they are unable to accurately reconstruct sections of the OR which are vital for utilisation of tractography techniques in clinical practice.

Analysis of ROC curves (Fig. 4A) showed that the CSD tractography was significantly more accurate (greater AUC and *J*) than the B&S tractography (Fig. 4B) when using the Julich atlas as a gold standard. The median normalized SDI threshold that resulted in a FPR of 2.1% (Table 1) was used to compute the final SDIs (Fig. 2C and D). Although this was somewhat subjective, it resulted in median FPRs (Table 1) that were consistent with previous ROC analyses [6] of OR tractography, thus allowing for comparison of sensitivities, tract volume and the anterior position of Meyer’s loop (Tables 1 and 2). The median sensitivities of the final CSD tracts were significantly higher than the B&S tracts. It should be noted that these tractography algorithms have a maximum sensitivity of less than 100% for any threshold greater than zero.

**Fig 5:**
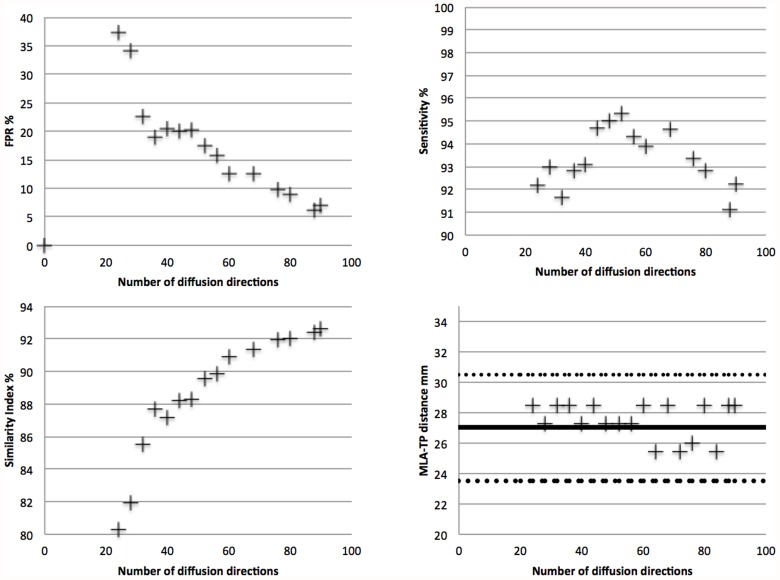
Dependence of OR tractography results on the number of diffusion directions in a single subject. Tractography was performed on a single subject from the human connectome project (www.humanconnectomeproject.org). The CSD tractography was performed on pre-processed 90 direction diffusion data, with a b-value of 3000 and 1.25mm isotropic voxel resolution, subsampled between 20 and 88 directions. The sensitivity, specificity and similarity indices were all computed using the fully sampled DWI data as the gold standard. All MLA-TP distances were within one standard deviation (dashed lines) of the median dissection distance (solid line).

A limitation of the study is that choosing the LGN as the seed ROI may have biased the results in favor of the CSD calculations, since previous results suggest seeding within the OR is optimal for diffusion tractography [6,16]. However, we believe this is compensated for by the fact that the LGN seed was directly taken from a freely available public atlas within FSL [24,25] and automatically registered to each subject’s scans. In addition, our results compare favorably to previous tractography results (Tables 1 & 2). Another limitation is the lack of a true “gold standard” to compare tractography results against. Our use of the Julich probabilistic atlas thresholded at 10% is far from ideal, which is why the position of Meyer’s loop was used as a comparison to previously published dissection results [2]. Further studies could be conducted comparing our methods against larger histological studies or more data-intensive DWI acquisition protocols. An example of how the number of directions can influence the tractography results is given in Fig. 5. It can be seen that as the number of diffusion directions was degraded from 90 to 20, the position of Meyer’s loop was stable within one standard deviation, the sensitivity remained above 80%, however the FPR increased significantly from 7 to 35%. For all probabilistic tractography algorithms it is difficult to determine an objective threshold [6] to distinguish between streamlines which are part of the fiber population of interest and those which are false positives (the “connectivity threshold”) [19,35,36]. Our results make use of an ROC procedure [6] as a best possible attempt to determine an objective and optimal threshold for each algorithm before comparing results. As can be seen in Figs. 2C and D, some false positive voxels clearly outside the OR are still present. Our use of an anterior coronal exclusion plane to exclude streamlines extending anteriorly outside the OR may influence results but was consistent with previous studies [6]. A more judicious use of exclusion and termination masks could prevent this but would be difficult to implement in an automated post-processing scheme. A final limitation is that it is not definitively known if the chosen b-value (3000 s/mm^2^) favoured one of the multifibre models, even though it should increase the angular contrast for both [19,21].

The more favorable CSD results should be considered only in the context of this acquisition protocol, post-processing scheme and publicly available open source technology. It may be that future developments allowing for smaller voxel resolution, improved FOD calculations and tracking algorithms may produce different results.

## Conclusion

This study showed that accurate CSD and B&S probabilistic tractography of the OR is possible with fully automated seeding and tracking algorithms using data acquired in a clinically feasible time frame. The data acquisition could readily be incorporated into a clinical MRI protocol with minimal impact to the patient. Moreover, our results showed that using CSD to compute local fiber orientations yielded improved tractography performance over the popular B&S model. CSD tractography was significantly faster, more sensitive, more accurate and in closer agreement with dissection studies than B&S tractography.

## Supporting Information

S1 Table List of Subjects. GBM = Glioblastoma multiforme.
(DOCX)
