# Supplementary material for: Probabilistic MRI Tractography of the Optic Radiation Using Constrained Spherical Deconvolution: A Feasibility Study
Source: PLoS One. 2015 Mar 5;10(3):e0118948. doi: 10.1371/journal.pone.0118948 (PMC4351098; doi:10.1371/journal.pone.0118948)
Supplement: S1 Table — GBM = Glioblastoma multiforme. (DOCX) [file pone.0118948.s001.docx]

| **Subject** | **Gender** | **Age** | **Diagnosis / Findings on MRI** | **Hemisphere chosen** |
| --- | --- | --- | --- | --- |
| S1 | F | 59 | Posterior superior sagittal sinus meningioma | Left |
| S2 | F | 41 | Right mesial temporal sclerosis | Left |
| S3 | M | 31 | Right mesial temporal sclerosis | Left |
| S4 | F | 47 | Residual pituitary tumour (transsphenoidal resection) | Left |
| S5 | M | 62 | Gliosis/demyelination of right supracerebellar peduncle and adjacent lateral ventricles | Left |
| S6 | M | 33 | Normal study | Left |
| S7 | F | 42 | Post-operative changes, right cerebellopontine angle meningioma resection | Left |
| S8 | M | 33 | Recent surgery for right frontoparietal glioma (known recurrent GBM) | Left |
| S9 | M | 30 | Normal study | Left |
| S10 | F | 39 | Stable right frontoparietal glioma, partially resected | Left |
| S11 | M | 64 | Previous right frontal lobe resection | Left |
| S12 | M | 28 | Bilateral demyelination | Right |
| S13 | F | 66 | Residual tumour, left cerebellopontine angle meningioma resection | Right |
| S14 | M | 45 | Simple pineal cysts | Right |
| S15 | M | 77 | Left parietooccipital tumour (consistent with GBM) | Right |
| S16 | M | 70 | Prior left superior frontal microhaemorrhage | Right |
| S17 | F | 52 | Residual left parietooccipital tumour | Right |
| S18 | F | 40 | Left parietal cystic lesion, post-craniotomy | Right |
| S19 | M | 31 | Post-operative changes, left cerebellar resection.  Previous midline 4^th^ ventricular mass resection | Right |
| S20 | F | 39 | Post-operative changes, left hemisphere gliosis and atrophy. Previous left frontal and parietal craniotomy | Right |

S1 Table.
